# Risk of Injury in Physically Active Students: Associated Factors and Quality of Life Aspects

**DOI:** 10.3390/ijerph17072564

**Published:** 2020-04-08

**Authors:** Elżbieta Sieńko-Awierianów, Monika Chudecka

**Affiliations:** Institute of Physical Culture Sciences, Faculty of Physical Education and Health, University of Szczecin, 71-065 Szczecin, Poland; elzbieta.sienko-awierianow@usz.edu.pl

**Keywords:** physical activity, risk of injury, quality of life, hypermobility, pain

## Abstract

Background: The aim of this study was to assess the potential factors of hypermobility and pain threshold on the risk of injury in physically active students and to verify which domains of quality of life are rated lower by young people with a history of injuries. Methods: The study included 278 students (138 women and 140 men) who regularly undertake physical activity. Anthropometric measurements, body composition, pain threshold, incidence of hypermobility syndrome, information on the history of injuries to the locomotor system, and the quality of life of the study participants were collected. Results: In the group studied, hypermobility and pain threshold had a statistically significant related on the risk of injury. Participants with a history of injuries had lower scores for an individual’s overall perception of their own health and the physical domain. There were also significant differences in the psychological domain of the quality of life between males and females with a history of injuries. Conclusion: In the studied group, the risk of injuries was related to diagnosed hypermobility and pain threshold measured on the lower limbs. The study also showed that people with a history of injuries had statistically significantly lower scores in the individual general perception of their own health and in the physical domain. Gender had a significant impact on the quality of life of people with injuries.

## 1. Introduction

Joint hypermobility (JH) is a condition where joints can move beyond the normal range of movement [1,2,3]. The terms joint hypermobility syndrome (JHS) and benign hypermobility joint syndrome (BHJS) refer to a condition where four or more joints may move outside their physiological range of movement [1,4]. JHS is often diagnosed clinically using the Beighton score [5].

Symptomatic and asymptomatic joint hypermobility is caused by heritable changes in proteins, which result in the laxity of connective tissue. This affects the stability of joint capsules and the extendibility of ligaments and tendons [6,7,8]. Joint hypermobility syndrome is a common condition, which has a significant impact on the quality of life of those it affects [9]. Its prevalence depends on many individual features, such as age, sex, ethnicity, and race [8,10]. The prevalence of joint hypermobility in children and adults is estimated at between 2% and almost 65% [4]. The divergence of results is mainly due to the various methods of evaluation (Beighton score or Brighton criteria), the differences between the populations studied, and the fact that joint hypermobility is highest during early childhood and continues to decrease during adolescence and adult life [4,6,7,11,12,13]. Regardless of the criteria used and the age of the population studied, joint hypermobility is more common in women than in men [4,14,15]. For example, Russek and Errico [15] found prevalence rates of 36.7% in women and 13.7% in men, while Reuter and Fichthorn [4] found prevalence rates of 16.2% in women and 8.7% in men.

Many people with hypermobility remain symptomless all their lives. People with joint hypermobility syndrome may have a predisposition to certain sports, such as ballet or dancing [4]. However, they may be at higher risk of injury to the musculoskeletal system, for instance, through sports-related injuries to the ankle, knee, and shoulder [16,17,18]. JHS is gaining increased attention as a potential source of pain and injury [15].

Pain is a specific sensation which has a protective role and warns us of danger. Pain informs athletes of the limits of their body and is part of the sports experience [19]. The pain tolerance of competitors seems to vary depending on the discipline [20]. For example, endurance-based sports are associated with improved pain inhibition and strength-based sports are associated with reduced pain sensitivity [21,22] Physical exercise results in changes in sensitivity to pain [23]. The eccentric stretching group showed a higher pressure pain threshold (PPT), determined using a pressure algometer, after exercise than the non-eccentric group [24].

There are many studies on the relationship between hypermobility or pain threshold and the risk of injury, but they focus mainly on professional contact and non-contact sports players and dancers [1,4,18,20,25,26,27,28,29,30,31].

However, there is limited information on whether such dependencies also occur in the demographic of young non-athletes who regularly have some form of physical activity [32,33,34,35]. Hence, the aim of this study was to assess the influence of hypermobility and pain threshold on the risk of injury in physically active students.

Physical activity has a positive impact on quality of life [36,37,38]. However, sports injuries may result in certain domains of the quality of life being rated lower. Therefore, a further aim of the study was to verify which domains of the quality of life of students who regularly undertake physical activity are rated lower as a result of the injuries suffered.

## 2. Materials and Methods

### 2.1. Participants

The study was performed on 278 students (138 women and 140 men) aged between 19 and 26 (20.98 ± 1.59) at the two largest universities in West Pomerania Province: the University of Szczecin and West Pomeranian University of Technology, Szczecin. The criteria for inclusion in the study were regular participation (at least twice a week) in selected forms of physical activity, namely aerobic, aerobic-strength, or cardio classes, at fitness centers in at least the previous two years and a BMI of between 18.5 and 29.99 [39]. These fitness classes require rapid acceleration, deceleration, and direction changes that exert significant forces on articular and periarticular structures. Each of the participants gave written consent before participating in the study. The study was approved by the Local Ethics Committee of the Pomeranian Medical University (Ethics Committee of the Pomeranian Medical University; No. 10/KB/VI/2018). The study was performed in compliance with the Declaration of Helsinki.

### 2.2. Measurements

Anthropometric measurements of height and weight were taken for the study participants. Moreover, body composition, namely PBF (Percentage Body Fat), FFM (Free Fat Mass in kg), and MM (Muscle Mass in kg), and BMI were assessed using the bioimpedance method with the use of an inBody 170 analyzer. For the purposes of the study, information on the chronological age, type, and frequency of physical activity at fitness centers and a history of injuries to the locomotor system (in accordance with the Orchard Sports Injury Classification System (OSICS) 10.1. Plus) [40] was gathered.

Pressure pain threshold was assessed using a pressure algometer manufactured by Quirumed. Algometry is a quantitative method for the assessment of tenderness that is commonly used in clinical practice [41]. PPT is defined as the minimal amount of pressure where the pressure sensation first becomes painful [42]. The PPT of the participants was measured by gradually increasing the pressure exerted by the head of the algometer, which was placed at an angle of 90 degrees, with a speed of 100 g/s until the pressure sensation first became painful (the participants were instructed to say “stop” when they felt a painful sensation). A mean for three measurements was then calculated and used for the main analysis. A 30-s rest between each measurement was allowed. PPT was measured bilaterally at two sites: the first dorsal interossei of the right and left hand and the tibialis anterior muscle.

The participants were tested for joint hypermobility. The main scale for the assessment of joint hypermobility is the modified Beighton score [5,43]. The score is a set of five simple maneuvers and is calculated by adding the points obtained for each maneuver. Joint hypermobility is present if at least 4 out of 9 points are scored. The diagnostics (of the right and left side of the body) include: passive dorsiflexion of the fifth metacarpophalangeal joint (MCP) to at least 90 degrees; apposition of the thumb to the forearm; hyperextension of the elbow to at least 10 degrees; hyperextension of the knee to at least 10 degrees; and forward flexion with the hands flat on the floor and the knees extended [44].

Quality of life was assessed using the standardized WHOQoL-BREF questionnaire, an abridged version of the World Health Organization Quality of Life questionnaire, which has been adapted for the Polish language, cultural and psychometric conditions [45]. The WHOQoL-BREF questionnaire comprises 26 questions. It is used in clinical practice to assess the quality of life of healthy and sick people and focuses on the following domains of quality of life: physical, psychological, social relationships, and environment. The following aspects are assessed individually: in the physical domain (Domain 1 (DOM1)): activities of daily living, dependence on medicinal substances and medical aids, energy and fatigue, mobility, pain and discomfort, sleep and rest, and work capacity; in the psychological domain (Domain 2 (DOM2)): body image and appearance, negative feelings, positive feelings, self-esteem, spirituality, religion, personal beliefs, thinking, learning, memory, and concentration; in the social relationships domain (Domain 3 (DOM3)): personal relationships, social support, and sexual activity; and in the environment domain (Domain 4 (DOM4)): financial resources, freedom, physical safety and security, health and social care (accessibility and quality), home environment, opportunities for acquiring new information and skills, participation in and opportunities for recreation and leisure activities, physical environment (pollution, noise, traffic, and climate), and transport. Moreover, the WHOQOL-BREF questionnaire includes two items that are assessed separately: Question 1 (WHO1), which asks about an individual’s overall perception of quality of life, and Question 2 (WHO2), which asks about an individual’s overall perception of their health. Questions are answered on a five-point scale (1–5). A maximum of 20 points can be scored in each domain. Domain scores are scaled in a positive direction (i.e., higher scores denote higher quality of life). Quality of life scores for particular domains were expressed as means, calculated in accordance with the key and guidelines provided by the authors [45,46].

### 2.3. Statistical Analyses

The results obtained were analyzed statistically. Distributions were examined using the Shapiro–Wilk test, which indicated that some variables deviated from a normal distribution (they were lognormal). The collected data were analyzed using the following statistical analyses: descriptive statistics, the Spearman’s rank correlation, and the Mann–Whitney U test.

The accepted level of significance was defined as 𝑝 < 0.05. Each parameter was characterized by the sample size, arithmetic mean, and standard deviation. Spearman’s rank correlation coefficient was used to determine correlations between the parameters analyzed (age, training experience in years, frequency of training per a week, morphological treats, BMI, body composition, hypermobility, and pain threshold) and the risk of injury to participants (also separately for men and women). The significant differences regarding the variables in WHOQoL-BREF questionnaires between participants with a history of injuries and those without a history of injuries, as well as between male and female participants with a history of injuries, were evaluated by means of the Mann–Whitney U test.

Statistical results were obtained using STATISTICA PL v.13.1 software (Statsoft, Krakow, Poland).

## 3. Results

Table 1 shows the descriptive characteristics of the group studied, with a breakdown by sex. The following data are presented: chronological age, length of training experience, frequency of training at a fitness centers, morphological parameters (height and weight), BMI, body composition parameters (PBF, FFM, and MM), hypermobility scores, and pain threshold values.

Of the 278 physically active students participating in the study, 113 students had a history of injuries (n = 113, 40.65%), including 49 women (n = 49, 35.51%) and 64 men (n = 64, 45.71%). On the basis of the data gathered, the Spearman’s rank correlation coefficient was calculated and related variables on the incidence of injury to the locomotor system in the study participants were analyzed. Two of the features analyzed, namely the presence of hypermobility (i.e., a score of at least 4 on the Beighton scale) and pain threshold measured on the lower limbs, were found to be statistically significantly related to the incidence of injury to the locomotor system in the study participants (Table 2).

In addition, a correlation between the parameters analyzed and the risk of injury was determined separately for men and women. An analysis of the Spearman’s rank correlation coefficient results carried out separately for men and women showed a statistically significant correlation between hypermobility and the risk of injury (*p* < 0.000) in both groups. A correlation was also observed between PPT 2 and the incidence of injury in the group of men (*p* < 0.027) and in the group of women (*p* < 0.049).

The next step was to analyze the results obtained from the WHOQoL-BREF quality of life questionnaire for students with a history of injuries and those without a history of injuries.

The results obtained from the WHOQoL-BREF questionnaires for participants with a history of injuries and those without a history of injuries, as well as intergroup differences, are presented in Table 3.

Statistically significant differences in the general health and physical health domains were found between these two groups. The level of satisfaction in the two domains was statistically significantly higher in the study participants without a history of injuries.

The next stage of the statistical analysis focused on determining whether there are any differences between men and women in the group of participants with a history of injuries (Table 4). Statistically significant differences were found between men and women in the psychological domain. The level of satisfaction in the domain was statistically significantly higher in male participants with a history of injuries.

## 4. Discussion

Diagnosis of hypermobility and its potential relation with the risk of injuries is an important research question, especially for physically active people. Our studies on young women and men who regularly undertake physical activity have shown that hypermobility is related in a statistically significant way to the risk of injury to the locomotor system in both sexes. The obtained results confirm the research of some authors who showed a similar relationship among athletes practicing competitive sports [4,17,18,25,26,27,28,33,34,37,47,48,49]. However, hypermobility should not lead to abandoning physical activity. If hypermobility is suspected, it is important to correctly diagnose and modify the training program (introduction of exercises to strengthen and stabilize the joints) in order to minimize the risk of injury [18,50,51].

Interestingly, contrary to the results of our research, some studies involving women with JHS, on dance students [52], NCAA lacrosse players [31], and elite female soccer players [53], did not show an increased risk for musculoskeletal injuries.

The second variable which was found to be statistically significantly related to the risk of injury was pain threshold measured on the lower limbs. The literature on the subject includes numerous studies which confirm that physically active people have a higher pain threshold and tolerance compared with physically inactive people [20,54,55]. On the one hand, a higher pain threshold and tolerance may help succeed in sports. On the other hand, people with lower pain sensitivity face the risk of overstrain, which is harmful to health and often leads to injury [56]. Failure to properly assess the risks associated with tissue overstrain caused by ignoring pain may be one of the key determinants of injury [57]. Our studies confirm the correlation between the risk of injury and pain threshold, although this was only on the lower limbs.

Physical activity is an important determinant of quality of life, and quality of life is a key factor which motivates people to be physically active [58,59]. Numerous studies analyzing the correlation between quality of life and physical activity have shown that physically active people rate certain domains of quality of life higher compared with physically inactive people. Some studied pointed out that people with disabilities who exercise regularly had higher quality perceptions than normal people with lower levels of physical activity [36,59,60,61,62,63]. While participation in sporting activities has multifaceted benefits in each domain of quality of life, it also carries a potential risk of injury [35,64]. Sports injuries may have long-term physical and psychological consequences and may have an impact on health-related quality of life and life satisfaction [65]. Our studies on young people who regularly undertake physical activity have shown that people with a history of sports injuries had lower quality of life scores in all the domains in the WHOQoL-BREF questionnaire. However, statistically significant differences between the two groups of study participants were only found in the case of an individual’s overall perception of their own health and the physical domain.

Variables which have an impact on particular domains of quality of life also include sex [66,67,68]. In a study on the quality of life of medical students in China carried out using the WHOQoL-BREF questionnaire, Zhang et al. [66] showed that male students had significantly higher quality of life scores in the psychological domain compared with female students, which, according to the authors, was because women are more emotional and more vulnerable to the pressure they are under. Similar conclusions have been drawn by other authors [69,70]. Our studies also showed that the level of satisfaction of female participants in the psychological domain was statistically significantly lower compared with men. Numerous studies have found that women have higher quality of life scores in the social relationships domain than men. Studies show that women cope better than men with different relations (personal relationships, social support, and sexual activity) [66,69,70,71]. Our research has not confirmed such gender differences in the social domain.

The limitation of this study consists in the fact that data came from only one region of Poland. Prospective studies should include data from the whole country, especially with studies regarding the quality of life. Our research involved physically active young people with normal body weight. Therefore, further research seems to be necessary to determine the relationship among hypermobility, pain threshold, and sports injuries in physically active people in different age groups and in people with different BMI levels (including those who are overweight and obese).

## 5. Conclusions

Our research is an important contribution to the research on the relationships among hypermobility, pain threshold, and risk of injury. In the studied group, hypermobility influenced the risk of injuries. Reduced sensitivity to pain may expose people to dangerous levels of overexertion, often leading to injuries, which was confirmed by the correlation between the pain threshold measured on the lower limbs and the risk of injury in physically active young people. This study also showed that people with a history of injuries achieved statistically significantly lower values in the individual general perception of their own health and in the physical domain. Gender had a significant impact on the quality of life of people with injuries. Female students with a history of injuries had significantly lower quality of life scores in the psychological domain compared with male students, since women are more emotional. The modern lifestyle, which promotes undertaking various forms of physical activity, implies the need for further research on factors influencing the risk of injury in the general population. Identifying these factors can help in finding adequate preventative action to minimize injuries and improve quality of life.

## Figures and Tables

**Table 1:** Descriptive characteristics of the group studied, with a breakdown by sex.

Factors	Women				Men			
	M	SD	Min.	Max.	M	SD	Min.	Max.
Age (years)	20.73	1.29	19.00	24.00	21.24	1.89	19.00	26.00
Training experience (years)	3.53	1.25	2.00	7.00	4.35	1.31	2.00	9.00
Freq. of tr. (per week)	3.30	1.22	2.00	6.00	3.96	1.35	2.00	7.00
Height (cm)	168.37	5.54	158.00	185.00	180.28	6.35	165.00	193.00
Weight (kg)	62.14	6.64	49.30	85.00	79.03	7.89	63.70	99.20
BMI (kg/m^2^)	21.89	2.06	18.30	29.10	24.31	2.03	20.20	29.80
PBF (%)	25.72	5.27	14.80	40.40	15.77	5.85	4.70	33.20
FFM (kg)	45.37	4.61	35.60	57.60	66.76	7.20	49.10	85.60
MM (kg)	22.40	3.87	14.50	32.00	37.17	5.69	12.20	48.90
Hypermobility	4.32	1.78	0.00	9.00	2.72	1.94	0.00	7.00
PPT 1 (N/s)	9.55	3.19	3.00	17.00	10.95	3.52	5.00	20.00
PPT 2 (N/s)	11.32	3.23	5.00	20.00	13.91	3.9	6.20	23.00

**Table 2:** Spearman’s rank correlation coefficient.

Factors	R	*p*
Age (years)	0.042270	0.483
Training experience (years)	−0.034231	0.569
Freq. of tr. (per week)	0.018801	0.754
Height (cm)	0.068398	0.256
Weight (kg)	0.057591	0.339
BMI (kg/m^2^)	0.032220	0.593
PBF (%)	−0.012275	0.839
FFM (kg)	0.058547	0.331
MM (kg)	0.079127	0.188
Hypermobility	0.270531	0.000 ***
PPT 1 (N/s)	0.070674	0.240
PPT 2 (N/s)	0.135205	0.024 *

* Statistically significant for 𝑝 < 0.05; *** Statistically significant for *p* < 0.001.

**Table 3:** Medians, 25th and 75th percentiles, means (M), and standard deviations (SD) for the Quality of Life-Brief Questionnaire (WHOQoL-BREF) and intergroup comparisons (with and without a history of injuries) using the Mann–Whitney U test.

WHOQoL-BREF Factors	Injury/No Injury	M ± SD	25th	Median	75th	*p*
Overall quality of life	Injury	4.05 ± 0.61	4.00	4.00	4.00	0.234
	No injury	4.16 ± 0.56	4.00	4.00	5.00	
General health	Injury	3.74 ± 0.81	3.00	4.00	4.00	0.003 *
	No injury	4.04 ± 0.73	4.00	4.00	5.00	
**WHO Domain**						
Physical health	Injury	15.25 ± 2.12	14.28	14.86	16.57	0.004 *
	No injury	16 ± 1.92	14.86	16.00	17.14	
Psychological	Injury	15.52 ± 2.17	14.00	16.00	16.67	0.241
	No injury	15.87 ± 1.82	14.67	16.00	17.33	
Social relationships	Injury	16.41 ± 2.50	14.67	17.33	18.67	0.735
	No injury	16.55 ± 2.50	16.00	16.00	18.67	
Environment	Injury	15.03 ± 1.89	14.50	15.00	16.50	0.052
	No injury	15.47 ± 1.67	14.50	15.50	16.50	

* Statistically significant for 𝑝 < 0.05.

**Table 4:** Medians, 25th and 75th percentiles, means (M), and standard deviations (SD) for the Quality of Life-brief Questionnaire (WHOQoL-BREF), with a breakdown by sex using the Mann–Whitney U test.

WHOQoL-BREF Factors	Sex	M ± SD	25th	Median	75th	*p*
Overall quality of life	Women	4.10 ± 0.68	4.00	4.00	4.00	0.256
	Men	4.02 ± 0.56	4.00	4.00	5.00	
General health	Women	3.75 ± 0.90	3.00	4.00	4.00	0.768
	Men	3.73 ± 0.74	3.00	4.00	5.00	
**WHO Domain**						
Physical	Women	15.10 ± 2.44	13.14	14.86	16.57	0.470
	Men	15.36 ± 1.87	14.29	16.57	17.14	
Psychological	Women	14.77 ± 2.44	13.33	14.67	16.00	0.001 *
	Men	16.09 ± 1.75	15.33	17.33	17.33	
Social relationships	Women	16.76 ± 2.42	16.00	17.33	18.67	0.213
	Men	16.15 ± 2.55	14.67	17.33	18.67	
Environment	Women	15.36 ± 1.72	14.50	15.00	16.50	0.093
	Men	14.77 ± 1.98	14.00	16.00	16.50	

* Statistically significant for 𝑝 < 0.05.
